# Fuzzy weighted natural nearest neighbor based density peak clustering

**DOI:** 10.1038/s41598-025-34175-0

**Published:** 2026-01-20

**Authors:** Mingzhao Wang, Xiangzhong Chen, Juanying Xie

**Affiliations:** School of Artificial Intelligence and Computer Science, Shannxi Normal University, Xi’an, 710119 China

**Keywords:** Density peaks, Local density, Natural nearest neighborhood, Fuzzy weighted natural nearest neighbors, Clustering, Computer science, Information technology

## Abstract

DPC (density peaks clustering) algorithm has garnered widespread attention due to its novelty and superior performance. However, it is sensitive to the arbitrary *cutoff* distance, and its very efficient assignment strategy is prone to leading “domino effect”. Although FKNN-DPC and other variants addressed DPC’s limitations somewhat, the arbitrarily fixed number of neighbors to calculate the local density of a point will bring the bias, particularly for the dataset containing dense and sparse clusters simultaneously, resulting in the bias to cluster centers and that in the final clustering. To remedy these limitations, this paper proposes a novel Fuzzy Weighted Natural Nearest Neighbor based parameter-free Density Peak clustering algorithm named FWNNN-DPC. It proposes a novel local density of a point utilizing its natural nearest neighbors by assuming that there is at least one “true friend” for a point when its natural nearest neighborhood is empty. Furthermore, a novel divide-and-conquer assignment strategy is proposed, which assigns non-outliers and outliers to the most appropriate clusters utilizing natural nearest neighbors based shortest distance principle, and fuzzy weighted natural nearest neighbors based membership degree, respectively. Extensive experiments on benchmark datasets and the statistic test demonstrate that the proposed FWNNN-DPC outperforms DPC and its variants, and the typical clustering algorithms DBSCAN and K-means.

## Introduction

Clustering as an unsupervised machine learning focuses on grouping data points into several groups based on their similarities, to ensure the points within a group are similar to each other while those belonging to other groups are dissimilar^1,2^. It plays an important role in various fields, such as social sciences, biology, pattern recognition, information retrieval, etc.^3–7^. With the rapid growth of complex data types (e.g., graph and text data) in recent years, clustering research has been further extended to these domains with innovative methods: For example, Xie et al. proposed the Robust Graph Structure Learning under Heterophily (RGSL) method^8^, which targets heterophilic graph data by designing a high-pass filter to enhance node feature discriminability and an adaptive $\alpha$-norm to handle noise, effectively improving graph clustering robustness in non-homophilic scenarios; their subsequent work, Feature Personalized Graph Clustering (FPGC)^9^, introduced a “one node one model” paradigm, using a squeeze-and-excitation block to select cluster-relevant features and feature cross augmentation to capture low-order feature interactions, addressing the “missing-half” feature information utilization issue in traditional graph clustering. Furthermore, Hou et al.^10^ comprehensively summarized image clustering methods based on deep learning. For text data, Petukhova et al.^11^ integrated large language model (LLM) embeddings into text clustering, providing new insights for text data structuring.

There are various types of clustering algorithms, such as partitioning-based ones, like K-means^12^, hierarchical ones, like BIRCH (Balanced Iterative Reducing and Clustering using Hierarchies)^13^, density-based ones, like DBSCAN (Density Based Spatial Clustering of Applications with Noise)^14^ and KNN-BLOCK DBSCAN^15^, and grid-based ones, like WaveCluster^16^. Besides, ensemble clustering-aiming to improve result robustness by fusing multiple base clusterings-has become a key direction. For example, the Anchor-based Fast Spectral Ensemble Clustering (FSEC)^17^ selects high-quality anchors via Balanced K-means-based Hierarchical K-means (BKHK), adopts a fast K-nearest neighbors approximation to build anchor graphs; notably, it produces multiple base clusterings through one spectral embedding, greatly speeding up ensemble clustering for large-scale datasets. Furthermore, there emerged a simple framework aiming for clustering complex data^18^.

Among these, density-based clustering algorithms are particularly effective, due to their ability in detecting clusters of arbitrary shapes within a dataset. DPC (Density Peaks Clustering)^19^ as a novel density based clustering algorithm has been attractive with the past decade. It defines local density ${{\rho }_{i}}$ and relative distance ${{\delta }_{i}}$ of point *i* within a dataset, displays all points in a decision graph using local density $\rho$ and relative distance $\delta$ as *x*-axis and *y*-axis, respectively. Those points in the upper-right corner are density peaks with both relatively high local densities and relative distances, comprising cluster centers. The remaining points are assigned to the same cluster as their nearest neighbors with higher density. Although DPC is efficient and effective, its performance relies heavily on the *cutoff* distance $d_c$; moreover, its assignment strategy can lead to “domino effect”, i.e., once a point is assigned erroneously, there may be many subsequent points will be assigned erroneously. To address these issues, many variants of DPC have been proposed.

The first type variants aim to address the limitation of the local density definition of DPC. KNN-DPC (K-Nearest Neighbors optimized Density Peak Clustering)^20^ and FKNN-DPC (Fuzzy weighted K-Nearest Neighbors Density Peak Clustering)^21^ are this type of variants. They defined the local density of a point using its K-nearest neighbors. SFKNN-DPC (Standard deviation weighted distance and Fuzzy weighted K-Nearest Neighbors based Density Peak Clustering)^22^ further enhanced the local density definition by taking into account the specific contribution of each feature to the distance between points, so that the local density of a point can embody the true distribution of points within a dataset as far as possible. DPC-KNN (Density peaks clustering based on k nearest neighbors)^23^ incorporates K-nearest neighbors to define the local density of a point while introducing PCA (Principal Component Analysis) to reduce the dimensionality of points within a dataset, so as to enhance the efficiency of the DPC algorithm. SNN-DPC(Shared-nearest-neighbor-based clustering by fast search and find of density peaks)^3^ defines the local density through shared nearest neighbor similarity, so that the local density can embody the true distribution of points. BC-DPC (Density peaks clustering based on k-nearest neighbors and self-recommendation)^24^ defines balanced density to eliminate the density differences between different clusters. ADA-DPC (An adaptive clustering algorithm by finding density peaks)^25^ defined the local density of a point using the standard deviation of its K-nearest neighbors. DPC-CE (Density peak clustering with connectivity estimation)^26^ algorithm introduces a graph-based connectivity estimation strategy (CES) to estimate the connectivity between two local centers. It adjusts the distance between two local centers based on connectivity information and distance penalties derived from spatial distances, thereby defining a new local density. DPCSA (Density peaks clustering based on weighted local density sequence and nearest neighbor assignment)^27^ algorithm divides the local density into two components: a fixed k-nearest neighbor term and a weighted sequence term, to avoid the need for pre-specified parameters. SDW-DPC (Standard deviation weighted distance based density peaks clustering)^28^ algorithm proposes a standard deviation-weighted distance to instead the Euclidean distance used in DPC. The RNN-CFSFDP (Reverse-nearest-neighbor-based clustering by fast search and find of density peaks)^29^ algorithm redefines the local density for samples by combining the reverse nearest neighbors of the samples. The ANN-DPC (Adaptive nearest neighbor density peak clustering)^30^ algorithm introduces adaptive nearest neighbors to define the local density of a point and classify it as super-core, core, linked, or slave point. WANN-DPC (Weighted Adaptive Nearest Neighbor DPC)^31^ is proposed to address the limitations of ANN-DPC. It defines the new local density of a point by weighting the different contributions of its close and far neighbors.

The second type of variants of DPC focus on automatically identifying cluster centers. McDPC (Multi-Center Density Peak Clustering) algorithm^32^ obtains initial cluster centers through the distances between points and other high-density points. VDPC (Variational Density Peak Clustering) algorithm^33^ introduces a novel way of selecting representative points to obtain cluster centers. ADA-DPC^25^ proposed a way to detect the cluster centers automatically. CPF (Component-wise Peak-Finding)^34^ algorithm is not affected by spurious density maxima, thus enabling it to automatically determine the correct number of clusters. DPC-SNFC (Density peaks clustering algorithm based on superior nodes and fuzzy correlation)^35^ algorithm utilizes reverse nearest neighbors to identify the nearest point with higher density as the superior node, then applies fuzzy correlation to construct connected subgraphs, thereby automatically identifying the number of clusters without selecting cluster centers. ANN-DPC^30^ algorithm introduces the super-core point absorption technique and dependency vector to automatically identify appropriate cluster centers. WANN-DPC^31^ proposes a correction factor to detect cluster centers one by one, resulting its capacity to detect all cluster centers no matter from dense or sparse clusters.

The third type of variants focus on enhancing assignment strategies to reduce error propagation. Most of this kind of variants incorporate the idea of the K-nearest neighbors. For instance, RECOME (RElative COre MErge clustering algorithm)^36^ proposed a labeling strategy based on KNN graph for assigning points to the appropriate clusters; KNN-DPC^20^ and ADA-DPC^25^ assign points based on the idea of K-nearest neighbors; FKNN-DPC^21^ respectively utilized the idea of K-nearest neighbors and fuzzy K-nearest neighbors to assign non-outliers and outliers to the appropriate clusters. SFKNN-DPC^22^ proposed the divide and conquer assignment strategy based on its proposed weighted distance and the semi-supervised learning and the mutual K-nearest neighbor assumption. DPCSA^27^ algorithm employed boundary conditions to divide the allocation strategy into two stages, reducing allocation error propagation. RNN-CFSFDP^29^ algorithm utilized the nearest neighbor samples to detect the local distribution of samples, thereby improving the allocation strategy. ANN-DPC^30^ algorithm proposed a novel assignment strategy by combining adaptive nearest neighbors with breadth-first search and fuzzy-weighted adaptive nearest neighbors. CPF^34^ algorithm improved the assignment methodology by applying the density peak approach to the estimated density level sets. R-MDPC (Fast main density peak clustering within relevant regions via a robust decision graph)^37^ algorithm’s relevance-based allocation strategy can effectively identify complex cluster structures by grouping relevant points together. WANN-DPC^31^ introduces a two-step assignment strategy utilizing the nearest neighbor relationships and the weighted membership degrees, resulting its superiority over its peers.

The aforementioned three types of DPC variants are summarized in Table 1, where “$\checkmark$” indicates the presence of the related variation in the specific study, and “$\times$” means the absence.

**Table 1:** Summary of the available variants of DPC algorithm.

Algorithms	Improving local density	Automatically identifying cluster centers	Enhancing assignment strategy
KNN-DPC^20^	$\checkmark$	$\times$	$\checkmark$
FKNN-DPC^21^	$\checkmark$	$\times$	$\checkmark$
SFKNN-DPC^22^	$\checkmark$	$\times$	$\checkmark$
DPC-KNN^23^	$\checkmark$	$\times$	$\times$
SNN-DPC^3^	$\checkmark$	$\times$	$\checkmark$
BC-DPC^24^	$\checkmark$	$\times$	$\times$
ADA-DPC^25^	$\checkmark$	$\checkmark$	$\checkmark$
DPC-CE^26^	$\checkmark$	$\times$	$\times$
DPCSA^27^	$\checkmark$	$\times$	$\checkmark$
SDW-DPC^28^	$\checkmark$	$\times$	$\times$
RNN-CFSFDP^29^	$\checkmark$	$\times$	$\checkmark$
ANN-DPC^30^	$\checkmark$	$\checkmark$	$\checkmark$
WANN-DPC^31^	$\checkmark$	$\checkmark$	$\checkmark$
McDPC^32^	$\times$	$\checkmark$	$\times$
VDPC^33^	$\times$	$\checkmark$	$\times$
CPF^34^	$\times$	$\checkmark$	$\checkmark$
DPC-SNFC^35^	$\times$	$\checkmark$	$\times$
R-MDPC^37^	$\times$	$\times$	$\checkmark$
RECOME^36^	$\times$	$\times$	$\checkmark$

Although the aforementioned variants of DPC have addressed some of the limitations and enhanced the performance of DPC algorithm, most of them rely on the K-nearest neighbors for computing the local density of a point and improving assignment strategies. The fixed K value for all points within a dataset may lead to errors in capturing the actual distribution of points because different clusters in a dataset may have large various densities.

In order to address the limitations of DPC and its variants, a fuzzy weighted natural nearest neighbor based density peak clustering algorithm, referred to FWNNN-DPC, is proposed in this study. The new local density is proposed in FWNNN-DPC using the natural nearest neighbors of a point. The divide-and-conquer assignment strategy is put forward utilizing the natural nearest neighbors based minimum distance principle for non-outliers, and fuzzy weighted natural nearest neighbors for outliers. This new local density definition avoids the fixed *K* used in many DPC variants, enabling the local density of a point to embody the genuine distribution around it as far as possible. Furthermore, the new proposed assignment strategy ensures the remaining points are to be clustered as accurate as possible.

Specifically, the core contributions of this work are as follows: First, by introducing the natural nearest neighbor (NNN) mechanism, the proposed algorithm avoids the reliance on the pre-specified parameter $K$ that is common in most DPC variants, allowing it to dynamically adapt to the local structural characteristics of data. Second, a novel local density calculation method is developed based on the natural nearest neighbors of a point. This method more truly reflects the actual data distribution around each point. Third, a divide-and-conquer assignment strategy integrated with NNN is proposed: for non-outliers, the minimum distance principle based on natural nearest neighbors is adopted for accurate cluster assignment; for outliers, fuzzy weighting based on NNN is introduced. This strategy enhances the accuracy and robustness of clustering for remaining points.

The remaining structure of this paper is as follows: we first provide a brief introduction to DPC and FKNN-DPC algorithms. Then we present the proposed FWNNN-DPC algorithm in detail. Next, the proposed FWNNN-DPC algorithm is tested on both synthetic and real-world datasets, and compared with DPC and its variants, and the very famous K-means and DBSCAN algorithms. Furthermore, the statistical tests and ablation experiments are, respectively, carried out to demonstrate the superiority of the FWNNN-DPC, and the contributions of FWNNN-DPC. Finally, we summarize the advantages and limitations of the FWNNN-DPC algorithm and highlight future research directions.

## DPC and FKNN-DPC algorithms

### DPC algorithm

DPC algorithm was proposed based on the assumption^19^ that the ideal cluster centers should possess two properties: (1) the local density of a cluster center is higher than the local densities of its neighbors, and (2) a cluster center is distant any points with higher local densities. To find cluster centers with the aforementioned characteristics, DPC algorithm defines the local density ${{\rho }_{i}}$ of point *i* by Eq. (1):

1 $$\begin{aligned} {{\rho }_{i}}=\sum \limits _{j\ne i}{\chi \left( {{d}_{ij}}-{{d}_{c}} \right) }\end{aligned}$$

where ${{d}_{ij}}$ represents the Euclidean distance between points *i* and *j*, and ${{d}_{c}}$ is the *cutoff* distance defined in advance, and the $\chi \left( x \right)$ is defined as follows.

2 $$\begin{aligned} \chi \left( x \right) =\left\{ \begin{matrix}1,\text x<0 \\ 0,\text x\ge 0 \\ \end{matrix} \right. \end{aligned}$$

These definitions imply that the local density of a point is depended on ${{d}_{c}}$, though the influence of ${{d}_{c}}$ on the local density of a point diminishes as the number of points in the dataset increases. Therefore, for a small size of dataset, the DPC algorithm uses a kernel-based approach to calculate the local density of samples, as shown in Eq. (3).

3 $$\begin{aligned} {{\rho }_{i}}=\sum \limits _{j\ne i}{\exp \left( -{{\left( \frac{{{d}_{ij}}}{{{d}_{c}}} \right) }^{2}} \right) }\end{aligned}$$

This local density defined in Eq. (3) can help reduce the impact of the *cutoff* distance ${{d}_{c}}$ on the local density and further on the clustering results of the DPC algorithm. To detect the cluster centers satisfying the assumptions, DPC algorithm further defines the relative distance ${{\delta }_{i}}$ for point *i* in Eq. (4), representing the distance from point *i* to the nearest point *j* with higher local density than point *i*, while if point *i* obtains the maximal density, its relative distance is defined as large as possible.

4 $$\delta _{i} = \left\{ {\begin{array}{*{20}l} {\max _{j} \left( {d_{{ij}} } \right),} \hfill & {\rho _{i} = \max \left\{ {\rho _{j} |j = 1, \ldots ,n} \right\}} \hfill \\ {\min _{{j:\rho _{j} > \rho _{i} }} \left( {d_{{ij}} } \right),} \hfill & {otherwise} \hfill \\ \end{array} } \right.$$

The other key contribution of DPC is the decision graph which is constructed using local density as *x*-axis and relative distance as *y*-axis, so that each data point can be displayed in this two-dimensional space. The points in the upper-right corner with both higher local density and relative distance are density peaks, comprising *Cluster Centers*. They are far away from those in the bottom-left corner. After the cluster centers have been detected out, the non-density peaks are to be assigned to the same cluster as the nearest point of higher local density. This one-step assignment strategy results in the very high efficient of DPC.

### FKNN-DPC algorithm

Although DPC is an efficient and superiority clustering algorithm, its limitations are obvious, such as it utilizes various local density definitions for various datasets, and the clustering results is depended on the *cutoff* distance ${{d}_{c}}$, and the “domino effect” exists in it. To remedy the limitations in DPC, FKNN-DPC algorithm was proposed^21^. It utilizes the idea of K-nearest neighbors to define the local density of samples. Additionally, it incorporates K-nearest neighbors and fuzzy weighted K-nearest neighbors to define a new assignment strategy for assigning non-cluster center samples to appropriate clusters. The local density definition of FKNN-DPC is as follows:

5 $$\begin{aligned} {{\rho }_{i}}=\sum \limits _{j\in KN{{N}_{i}}}{\exp \left( -{{d}_{ij}} \right) } \end{aligned}$$

Where $KNN_i$ represents the K-nearest neighbor set of point *i* and ${{d}_{ij}}$ is the Euclidean distance between points *i* and *j*.

The other contribution of FKNN-DPC is that it proposes a two-step assignment strategy for assigning non-density peak points. This strategy partitions the remaining points into outliers and non-outliers. For non-outliers, it utilizes the breadth first search and the K-nearest neighbors to assign them to the appropriate clusters; while for outliers, it employs the idea of fuzzy weighted K-nearest neighbors. It calculates the membership degree of each outlier point to each cluster, and iteratively assign the point with the highest membership and updates the rest points’ memberships till all outliers are assigned to the appropriate clusters.

**Fig. 1:**
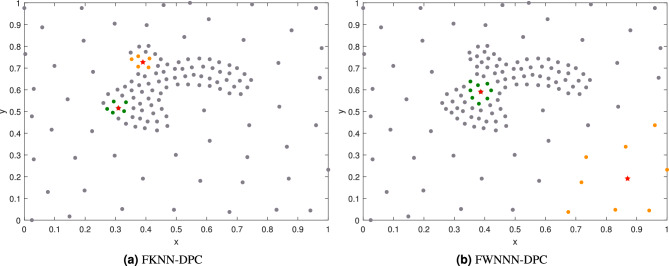
The cluster centers detected by FKNN-DPC and FWNNN-DPC algorithms for the dataset having clusters with significant density variation and overlapped in space.

Although FKNN-DPC algorithm effectively addresses DPC’s dependency on the *cutoff* distance and partially mitigates the “domino effect” caused by DPC’s assignment strategy, it still has its limitations. First, FKNN-DPC defines local density and allocates non-density peak points using K-nearest neighbors. But, in real scenarios, datasets may contain clusters with various densities. The use of a fixed *K* to calculate the local density of a point may lead to the bias in the local density of a point. Additionally, its local density definition may result in its failing in identifying the correct cluster centers of a dataset, particular it contains clusters with significant density variation but overlapped in space, further leading to more erroneously clustered points. As is illustrated in Fig. 1, where Fig. 1a illustrates the cluster centers identified by FKNN-DPC with *K*=5, and Fig. 1b is the results by FWNNN-DPC. It can be observed from Fig. 1a that the detected cluster centers by FKNN-DPC are all located in the dense cluster, failing to identify the cluster center in the surrounding sparse cluster. The FWNNN-DPC proposed in this study utilizes the natural nearest neighbors of a point to define its local density, enabling its detection of the cluster centers from dense and sparse clusters simultaneously, depicted in Fig. 1b.

## FWNNN-DPC algorithm

This section will introduce the proposed FWNNN-DPC algorithm in detail, including the basic idea of Natural Nearest Neighborhood (NNN) proposed in^38–40^, the new local density of a point and the new assignment strategy for assigning points to the most appropriate clusters, and the main steps of the FWNNN-DPC and its complexity analysis.

### Natural nearest neighborhood

The concept of Natural Nearest Neighborhood (NNN) is scale-free, and it does not need setting parameters for selecting neighbors for points^38^ . Depending on the real distribution of a dataset, the specific NNN can vary for each point.

The basic idea behind NNN is that the number of “true friends” of a person should be determined by the number of persons who consider this person as friends, rather than the number of persons that this person considers them as his friends. In this paper, the “true friend” of a point is equivalent to the number of its natural nearest neighbors. Moreover, it is assumed that each point should have at least one “true friend”, because when the most isolated person has at least one “true friend”, the society reaches a stable status^39^.

In a dataset, when the extreme outlier becomes one of the K-nearest neighbors of another point, there is a tendency for some points to have a greater number of neighbors than others. This is in accordance with the actual distribution of points within a dataset. This allows us to gain insights of the local information of a point. The concepts of natural nearest neighborhood and related concepts are described as follows.

Let $\boldsymbol{X=\left\{ {{x}_{1}},{{x}_{2}},\ldots ,{{x}_{n}} \right\} }\subset {\boldsymbol{{R}}^{m}}$ be a dataset, the Euclidean distance ${{d}_{ij}}$ is used to measure the distance between points *i* and *j*. Suppose parameter $k>0$, let $n{{n}_{k}}\left( {\boldsymbol{x}_{i}} \right)$ denote the *k*-th nearest neighbor of $\boldsymbol{{x}_{i}}$ in $\boldsymbol{X\backslash \left\{ {{x}_{i}} \right\} }$, we define the set of the *k*-nearest neighbors of $\boldsymbol{{x}_{i}}$ as $\boldsymbol{N{N}}_{k}\boldsymbol{\left( {{x}_{i}} \right) }$, which is represented as follows:

6 $$\begin{aligned} \boldsymbol{N{N}}_{k}\boldsymbol{\left( {{x}_{i}} \right) }=\cup _{j=1}^{k}\left\{ n{{n}_{j}}\left( \boldsymbol{{x}_{i}} \right) \right\} \end{aligned}$$

The set of *k*-reverse nearest neighbors of $\boldsymbol{{x}_{i}}$ referred to $\boldsymbol{RN{N}}_{k}\boldsymbol{\left( {{x}_{i}} \right) }$ is defined as the set of points $\boldsymbol{{x}_{j}}$ within the dataset $\boldsymbol{X}$ excluding $\boldsymbol{{x}_{i}}$ while $\boldsymbol{N{N}}_{k}\boldsymbol{\left( {{x}_{j}} \right) }$ containing $\boldsymbol{{x}_{i}}$. This can be expressed by follows:

7 $$\begin{aligned} \boldsymbol{RN{N}}_{k}\boldsymbol{( {{x}_{i}} )} =\{ \boldsymbol{{x}_{j}}\in \boldsymbol{X}\backslash \boldsymbol{ \{{x}_{i}\}}|\boldsymbol{{x}_{i}}\in \boldsymbol{N{N}}_{k}\boldsymbol{( {{x}_{j}} ) \}} \end{aligned}$$

The *k* natural nearest neighborhood of $\boldsymbol{{{x}_{i}}}$ denoted as $\boldsymbol{NN{N}}_{k}\boldsymbol{\left( {{x}_{i}} \right) }$ is defined as:

8 $$\begin{aligned} \boldsymbol{NN{N}}_{k}\boldsymbol{\left( {{x}_{i}} \right) }=\boldsymbol{N{N}}_{k}\boldsymbol{\left( {{x}_{i}} \right) }\cap \boldsymbol{RN{N}}_{k}\boldsymbol{\left( {{x}_{i}} \right) }\end{aligned}$$

It is obvious that, for $\forall \boldsymbol{{x}_{i}}\in \boldsymbol{X}$, there exists $\boldsymbol{NN{N}}_{n-1}\boldsymbol{\left( {{x}_{i}} \right) }=\boldsymbol{X\backslash \{{x}_{i}\}}\ne \varnothing$. The minimum integer $\lambda$ making $\boldsymbol{NN{N}}_{\lambda }\boldsymbol{\left( {{x}_{i}} \right) }\ne \varnothing$ for $\forall \boldsymbol{{x}_{i}}\in \boldsymbol{X}$ is called the natural eigenvalue of $\boldsymbol{X}$, and $\boldsymbol{NN{N}}_{\lambda }\boldsymbol{\left( {{x}_{i}} \right) }$ means the natural nearest neighborhood of $\boldsymbol{{x}_{i}}$, and is denoted as $\boldsymbol{NNN\left( {{x}_{i}} \right) }$^40^.

Let $\boldsymbol{NNN}_{\lambda }^{0}\boldsymbol{\left( X \right) }$ represent the dataset comprising points from $\boldsymbol{X}$ having the empty natural nearest neighborhood, that is:

9 $$\begin{aligned} \boldsymbol{NNN}_{\lambda }^{0}\boldsymbol{( X )}=\{\boldsymbol{{{x}_{j}}|{{x}_{j}}\in X} \text { and } \boldsymbol{NN{N}}_{\lambda }\boldsymbol{\left( {{x}_{j}} \right) }=\varnothing \} \end{aligned}$$

The natural eigenvalue $\lambda$ of $\boldsymbol{X}$ also represents the number of iterations for finding the natural nearest neighborhood $\boldsymbol{NNN\left( {{x}_{i}} \right) }$ for $\forall \boldsymbol{{x}_{i}}\in \boldsymbol{X}$. When there are no outliers or noises, the $\lambda$ is usually small. However, in some extreme cases, $\lambda$ may be the possible maximum value of *n*-1.

To reduce the influence from outliers or noises on the final size of $\lambda$, a threshold (=$\ln n+\ln \lambda$) is introduced to control the number of iterations when $\boldsymbol{NNN}_{\lambda }^{0}\left( {\boldsymbol{X}} \right)$ remains unchanged, i.e., $\boldsymbol{NNN}_{\lambda }^{0}\left( {\boldsymbol{X}} \right) =\boldsymbol{NNN}_{\lambda +1}^{0}\left( {\boldsymbol{X}} \right)$, during the iteration process^40^. However, under such case, there may exist some points, such as $\boldsymbol{{x}_{i}}$, whose $\boldsymbol{NNN\left( {{x}_{i}} \right) }$ is empty. This contradicts the original idea of the natural nearest neighborhood, where each point should have at least one “true friend”.

To address this limitation in^40^, which may lead to the empty of the natural nearest neighborhood of a point within a dataset, we set the number of the natural nearest neighbors of a point to be 1 when its natural nearest neighborhood is empty after calculating the natural nearest neighborhood for each point within a dataset.

The procedure for computing the natural nearest neighborhood for $\forall \boldsymbol{{x}_{i}}\in \boldsymbol{X}$ is described in Algorithm 1. Up to now, we can obtain the adaptive number of natural nearest neighbors for each point utilizing Algorithm 1, resulting in the nuanced assessment of the local neighborhood of a point within dataset, instead of the arbitrarily giving of the fixed number of the nearest neighbors for each point within a dataset, such as *K* in FKNN-DPC and other DPC variants based on *K*-nearest neighbors.

This natural nearest neighbor identification mechanism is inherently adaptive and does not require any user-specified parameter. As a result, it yields stable local density estimates for each point, as reflected in the local density definition in Eq. (10). Moreover, the use of natural nearest neighbors facilitates the assignment of non-density-peak points—non-outliers and outliers—to the most appropriate clusters. Since both non-outliers and outliers are assigned based on the adaptive neighborhood structure, the clustering process remains consistent and well-posed. This design ensures the stability and convergence of the proposed FWNNN algorithm.

**Algorithm 1:**
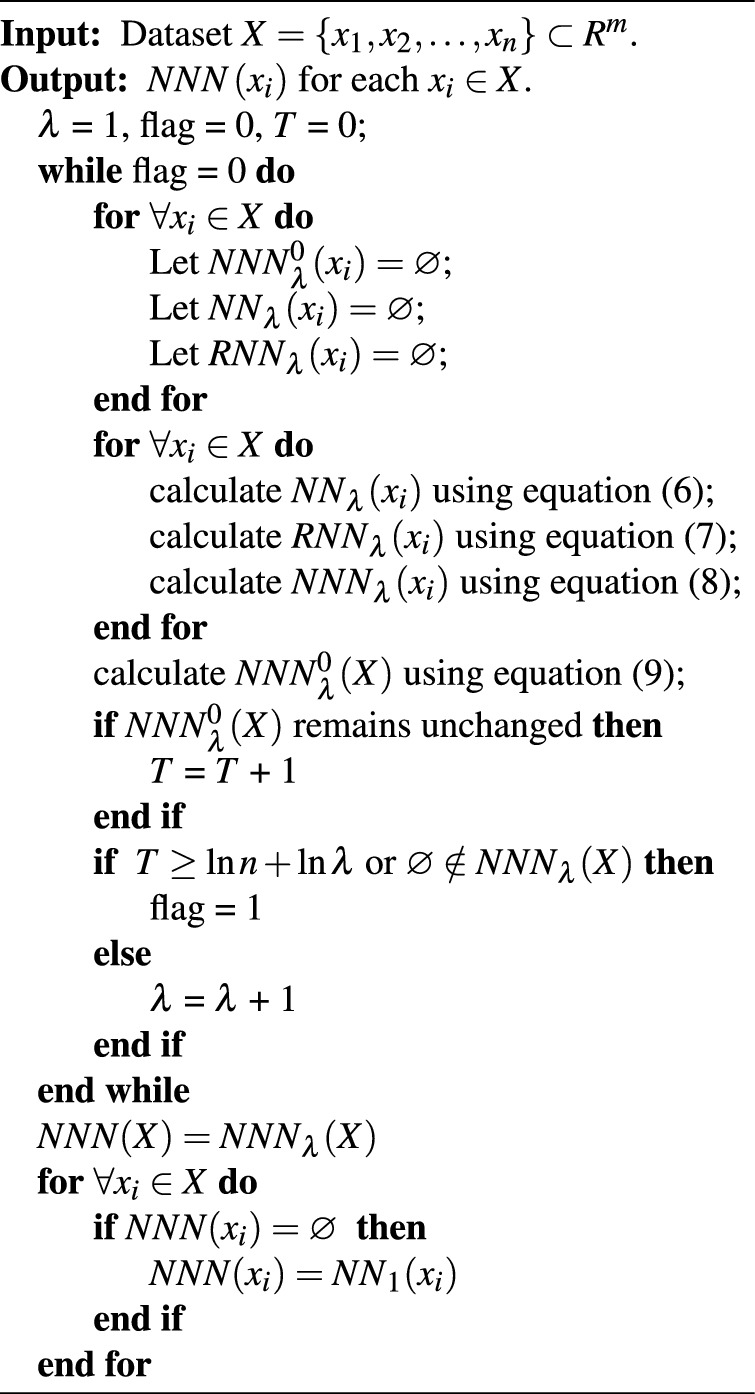
Finding the Natural Nearest Neighborhood for each point.

### Local density

After obtaining the natural nearest neighborhood of each point within a dataset, we define the local density $\rho _i$ of point *i* within the dataset as follows:

10 $$\begin{aligned} {{\rho }_{i}}=Normal\left( \left| \boldsymbol{NNN\left( {{x}_{i}} \right) } \right| +\sum \limits _{j\in \boldsymbol{NNN\left( {{x}_{i}} \right) }}{\left| \boldsymbol{NNN\left( {{x}_{j}} \right) } \right| } \right) + Normal\left( \sum \limits _{j\in \boldsymbol{NNN\left( {{x}_{i}} \right) }}{\exp \left( -{{d}_{ij}} \right) } \right) \end{aligned}$$

where, *Normal* is the max-min normalization, which normalizes data to fall in [-1,1]. The $\left| \boldsymbol{NNN\left( {{x}_{i}} \right) } \right|$ and $\left| \boldsymbol{NNN\left( {{x}_{j}} \right) } \right|$ represent the number of points in $\boldsymbol{NNN\left( {{x}_{i}} \right) }$ and $\boldsymbol{NNN\left( {{x}_{j}} \right) }$, respectively. The ${{d}_{ij}}$ is the Euclidean distance between points *i* and *j*. This definition will obtain the more precise local density $\rho _i$ of point *i*.

### Assignment strategy

Inspired by the assignment strategy used in FKNN-DPC^21^, FWNNN-DPC introduces its own divide-and-conquer strategy. Firstly, the non-density peak points are classified into outliers and non-outliers. Then the non-outliers and outliers will be respectively assigned to the most appropriate clusters as far as possible by utilizing the assignment strategies proposed in FWNNN-DPC in this paper. The outliers are defined by Eq. (11).

11 $$\begin{aligned} Outliers=\left\{ {{o}_{i}}|{{r}_{i}}^{\left| \boldsymbol{NNN\left( {{x}_{i}} \right) } \right| }>\tau ,\,i=1,\cdots ,n \right\} \end{aligned}$$

where, ${{r}_{i}}^{\left| \boldsymbol{NNN\left( {{x}_{i}} \right) } \right| }={{\max }_{j\in \boldsymbol{NNN\left( {{x}_{i}} \right) }}}\left\{ {{d}_{ij}} \right\}$, and $\tau =\frac{1}{n}\sum \limits _{i=1}^{n}{{{r}_{i}}^{\left| \boldsymbol{NNN\left( {{x}_{i}} \right) } \right| }}$. They are, respectively, the maximal natural nearest neighborhood radius of point *i*, and the average of it of all points within the dataset containing point *i*.

After identifying the outliers in a dataset, the *non-outliers* of it are composed of the remaining non-density peaks. FWNNN-DPC algorithm utilizes Algorithm 2 to assign non-outliers to the most appropriate cluster as far as possible. Then it employs Algorithm 3 to cluster outliers and the remaining unassigned points by Algorithm 2 to the most appropriate clusters.

It should be noted that Algorithm 2 first assigns the natural nearest neighbors of a cluster center to the same cluster with it, then assigns the unassigned non-outliers to its nearest cluster center by calculating the distances between them and all cluster centers, thereby assigning each non-outlier to its most appropriate cluster to mitigate the risk of consecutive assignment errors somewhat, i.e., the “domino effect”^20,21^ will be reduced to the least as far as possible. The Algorithm 2 is described as follows:

**Algorithm 2:**
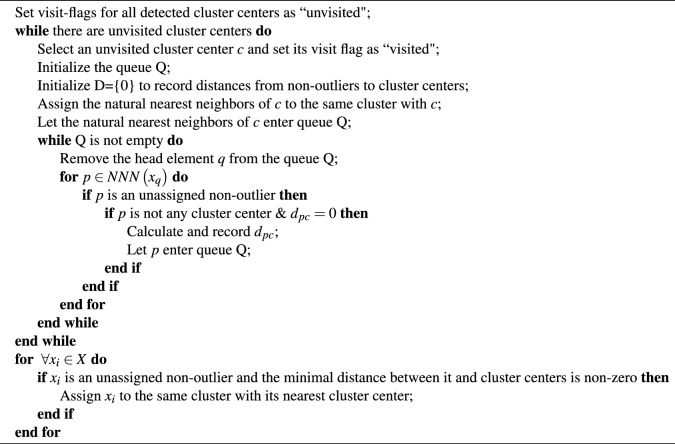
Assignment strategy for Non-outliers

The Algorithm 3 of FWNNN-DPC algorithm introduces fuzzy weighted natural neighbor idea and semi-supervised learning idea to assign the outliers and the remaining non-outliers by Algorithm 2. The key step of Algorithm 3 is to calculate the probability of a point belonging to a cluster. The point will be assigned to the cluster with the highest probability of belonging to that cluster.

We define $p_{i}^{c}$ in Eq. (12) to represent the membership probability of point *i* belonging to cluster *c*. The point *i* will be assigned to cluster *c* when $c = argmax \left\{ ~ p_i^c~ | ~c=1,\ldots , C~\right\}$.

12 $$\begin{aligned} {{p}_{i}}^{c}=\sum \limits _{j\in \boldsymbol{NNN\left( {{x}_{i}} \right) },{{y}_{j}}=c}{{{\gamma }_{ij}}\times {{w}_{ij}}} \end{aligned}$$

where, ${{w}_{ij}}=\frac{1}{1+{{d}_{ij}}}$ represents the similarity between points *i* and *j*, and ${{d}_{ij}}$ is the Euclidean distance between points *i* and *j*, and $\gamma _{{ij}} = \frac{{w_{{ij}} }}{{\sum\nolimits_{{l \in NNN\left( {x_{j} } \right)}} {w_{{lj}} } }}$ measures the ratio of ${{w}_{ij}}$ to the sum of the similarities between point *j* and its *natural* nearest neighbors. This aligns with the human intuition that neighbors are more likely to belong to the same category, serving as a direct quantification of the notion that “spatial proximity implies similarity”. The $\gamma _{ij}$ acts as the fuzzy weight of the similarity $w_{ij}$ to calculate the probability (fuzzy membership $p_i^c$) of point *i* belonging to cluster *c*. It measures the proportion of the similarity $w_{ij}$ between points *i* and *j* to that between point *j* and its all natural-nearest-neighbors. This definition indicates that the probability of point *i* belonging to cluster *c* (i.e., $p_i^c$) is not only determined by its natural-neatest-neighbors which have been assigned to cluster *c*, but also determined by its natural-nearest-neighbors’ natural-nearest-neighbors. Therefore, we can be say that this $p_{i}^{c}$ not only incorporates the similarity between points *i* and *j*, but also that of point *j* and its natural nearest neighbors, where point *j* is one of the natural nearest neighbor of point *i* and has been assigned to cluster *c*. It defines the fuzzy membership probability of point *i* to cluster *c* utilizing the fuzzy weighted similarity between points *i* and *j* by utilizing $\gamma _{ij}$ as the weight.

Furthermore, once a non-density peak *i* is assigned to its most appropriate cluster *c* by Algorithm 3, the membership degree of its natural nearest neighbor *j* to cluster *c*, i.e., $p_{j}^{c}$ is updated utilizing Eq. (13).

13 $$\begin{aligned} {{p}_{j}}^{c}={{p}_{j}}^{c}+{{\gamma }_{ij}}\times {{w}_{ij}} \end{aligned}$$

With these ideas in mind, the Algorithm 3 of FWNNN-DPC algorithm is described as follows:

**Algorithm 3:**
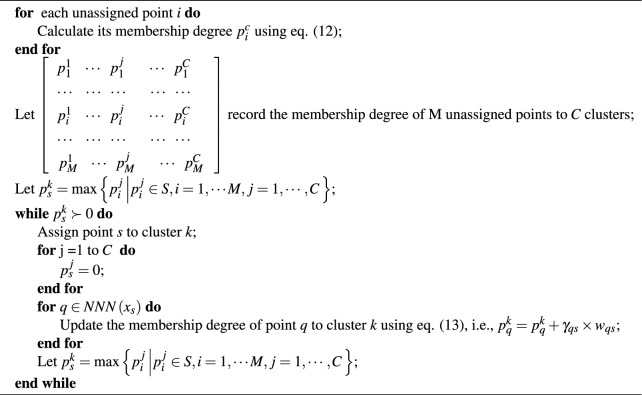
Assignment strategy for outliers.

### Main steps of FWNNN-DPC algorithm

With the aforementioned ideas, the main steps of the FWNNN-DPC algorithm are as follows:

Input: $$\boldsymbol{X=\left\{ {{x}_{1}},{{x}_{2}},\ldots ,{{x}_{n}} \right\} }\in {\boldsymbol{{R}}^{n\times m}}$$

Output: Clustering result C.

Step 1: Data preprocessing including filling missing values and data normalization;

Step 2: Utilizing Algorithm 1 to find the natural nearest neighbors and construct the natural nearest neighbor set $\boldsymbol{NNN\left( {{x}_{i}} \right) }$ for $\forall \boldsymbol{x}_i\in \boldsymbol{X};$

Step 3: Compute the Euclidean distance matrix between points;

Step 4: Calculate the local density ${{\rho }_{i}}$ and relative distance ${{\delta }_{i}}$ of point $\boldsymbol{x}_i\in \boldsymbol{X}$ in Eqs. (10) and (4), respectively.

Step 5: Construct decision graph using $\rho$ as *x*-axis and $\delta$ as *y*-axis;

Step 6: Select those points located in the upper right corner of the decision graph as cluster centers, which have both comparatively high local densities and relative distances, i.e., they are density peaks;

Step 7: Identify outliers utilizing Eq. (11) and non-outliers as well;

Step 8: Assign non-outliers utilizing Algorithm 2;

Step 9: Assign outliers and the remaining points unassigned by Algorithm 2 using Algorithm 3;

Step 10: If there are very few points unassigned, then assign them to the same cluster as the nearest point.

### Algorithm complexity analysis

Assume there are *n* points in the dataset, and each sample has *m* features. The space complexity of the DPC algorithm is $O\left( {{n}^{2}} \right)$ for storing distances between points. The FWNNN-DPC algorithm proposed in this paper also needs to store the distance matrix between points, resulting in the same space consumption as DPC did. In addition, FWNNN-DPC need calculate the natural nearest neighbors for each point, so it need space to store matrices for recording $\boldsymbol{N{N}}_{k}\boldsymbol{\left( {{x}_{i}} \right) }$, $\boldsymbol{RN{N}}_{k}\boldsymbol{\left( {{x}_{i}} \right) }$ and $\boldsymbol{NN{N}}_{k}\boldsymbol{\left( {{x}_{i}} \right) }$ of point *i*, and the matrix recording the natural nearest neighbors of each point, which are no more than $O(n \lambda )$, where $\lambda \ll n$. Additionally, the matrix $\boldsymbol{S}$ in Algorithm 3 for outliers and the unassigned points by Algorithm 2 need space to store, which is *O*(*MC*), where $M(<n)$ is the number of points to be assigned by Algorithm 3 and *C* is the number of clusters detected for a dataset by using the decision graph. Therefore, the space complexity of the FWNNN-DPC algorithm is the same as that of DPC algorithm’s, which is also $O\left( n^2\right)$.

The time complexity of FWNNN-DPC algorithm depends on the following five portions. (1) finding the natural nearest neighbor set for each point, and the time complexity is $O\left( {{n}^{2}}\ln n \right)$; (2) computing the distances between point, and the time complexity is $O\left( {{n}^{2}} \right)$; (3) calculating the local density $\rho _i$ for point *i*, which comprises finding the natural nearest neighbors of point *i* with the time complexity of $O\left( n \right)$, so, the time complexity of searching for the natural nearest neighbors of *n* points is $O\left( {{n}^{2}} \right)$; and the time complexity of calculating the local density of all points is $O\left( n \right)$, resulting in the total time complexity of calculating local densities of all points is $O\left( n^2+ n \right) \cong O\left( {{n}^{2}} \right)$; (4) calculating the relative distance $\delta$ for each point, and the time complexity is $O\left( {{n}^{2}} \right)$; (5) assigning non-density peaks, utilizing Algorithm 2 for non-outliers and Algorithm 3 for outliers and the remaining points of Algorithm 2, so the total time complexity of this step is $O\left( {{n}^{2}} \right)$. In conclusion, the time complexity of the FWNNN-DPC algorithm is $O\left( {{n}^{2}}\ln n \right)$, which is not as efficient as DPC algorithm.

So far, although numerous DPC variants have been proposed to improve its performance, relatively few studies have focused on enhancing its computational efficiency. Table 2 provides a detailed comparison of the time and space complexity of the proposed FWNNN-DPC with the original DPC and its representative variants FKNN-DPC and ANN-DPC.

**Table 2:** Time and space complexity of FWNNN-DPC and major baselines DPC, FKNN-DPC, and ANN-DPC.

Algorithms	FWNNN	DPC^19^	FKNN-DPC^21^	ANN-DPC^30^
Time complexity	$O(n^2\ln n)$	$O(n^2)$	$O(n^2)$	$O(n^2)$
Space complexity	$O(n^2)$	$O(n^2)$	*O*(*kn*)	$O(n^2)$

## Experimental results and analyses

To test the performance of the proposed FWNNN-DPC algorithm, we compare it to the classical algorithms and the DPC variants on synthetic and real-world datasets, where the real-world data are from the UCI machine learning repository^41^, and the synthetic datasets comprise the typical synthetic datasets for testing the clustering algorithms and the challenging ones created by us. Specifically, the FWNNN-DPC is compared to the very efficient and simple clustering algorithm K-means^12^, and the traditional density based clustering algorithm DBSCAN^14^, and the original DPC^19^, and the variations of DPC including FKNN-DPC^21^, DPCSA^27^, SFKNN-DPC^22^, SDW-DPC^28^, DPC-KNN^23^ and DPC-CE^26^. The results of K-means are the mean of its 20 runs, due to its results heavily depending on the initial partitions. The clustering results of algorithms are compared in terms of clustering accuracy (ACC), adjusted mutual information (AMI)^42^, and adjusted rand index (ARI)^42^. The upper bound of these metrics is 1, indicating the larger value of these metrics, the better is the clustering result. All algorithms were implemented in MATLAB 2018b, and the experiments were conducted in a Windows 11 operating system environment.

Because most of the algorithms in comparison need parameters, such as SDW-DPC and DPC algorithm requires specifying the *cutoff* distance as the specific percent of the distances between all points in ascending order; K-means requires the number of clusters *K* to be provided; DBSCAN needs two parameters: the radius of the neighborhood and the minimal number of points within the neighborhood; SFKNN-DPC and FKNN-DPC algorithms need providing *K* of the *K*-nearest neighbors. The DPC-KNN algorithm requires providing the percentage to the total number of points in a dataset to calculate the *K* of *K*-nearest neighbors. DPC-CE and DPCSA does not require any parameters. In order to exhibit the superiority of our FWNNN-DPC, we tuned parameters for each algorithm in comparison to the best through experiments, so as to compare the best performance of each algorithm in comparison to that of our FWNNN-DPC algorithm, which will further demonstrate the superiority of our FWNNN-DPC algorithm.

The real-world and Synthetic datasets used in experiments are listed in Table 3. The Cdataset1, Ellipse and Artil datasets displayed in Table 3 are generated by us, and the parameters used to generate them are shown in Tables 4, 5 and 6, respectively.

**Table 3:** Synthetic and real-world datasets used in experiments.

Real-world datasets	#instances	#Clusters	#Features	Sythetic datasets	#instances	#Clusters	#Features
Iris^41^	150	4	3	Compound^43^	399	2	6
Glass^41^	214	9	6	A3^44^	7500	2	50
Ecoli^41^	336	7	8	Target^45^	770	2	6
Libras movement^41^	360	91	15	Artil	10800	3	2
Dermatology^41^	366	34	6	Zelnik1^46^	299	2	3
Wdbc^41^	569	30	2	Complex9^25^	3031	2	9
Balance scale^41^	625	4	3	Pathbased2^47^	312	2	3
Pima-indians-diabetes^41^	768	8	2	Ellipse	8002	2	2
Yeast^41^	1484	8	8	Cdataset1	600	2	4
Spambase^41^	4601	57	2	Aggregation^48^	788	2	7
Waveform^41^	5000	21	3	Zelnik3^46^	266	2	3
Waveform (noise)^41^	5000	40	3	DIM512^49^	1024	512	16
Thyroid^41^	7200	5	3				
Letter^41^	20000	16	26

**Table 4:** Parameters for generating Cdataset1.

Datasets	Parameters	Cluster1	Cluster2	Cluster3	Cluster4
Cdataset1	Mean	[3, 10]	[3, 13]	[1.5, 7]	[5, 7]
	Covariance	$$\begin{bmatrix} 0.5 & 0 \\ 0 & 0.5 \end{bmatrix}$$	$$\begin{bmatrix} 0.8 & 0.1 \\ 0.1 & 0.8 \end{bmatrix}$$	$$\begin{bmatrix} 0.7 & 0.2 \\ 0.2 & 0.7 \end{bmatrix}$$	$$\begin{bmatrix} 1 & 0.2 \\ 0.2 & 1 \end{bmatrix}$$
	Points	300	100	100	100

**Table 5:** Parameters for generating Ellipse.

Datasets	Parameters	Cluster1	Cluster2
Ellipse	Mean	$t\in \left[ 0,\pi \right] x=\cos \left( t \right)$	[0, 0]
	Covariance	$y\in \left( \left( 2\sqrt{\left( 1-{{x}^{2}} \right) },3\sqrt{\left( 1-{{x}^{2}} \right) } \right) \bigcup \left( -3\sqrt{\left( 1-{{x}^{2}} \right) },-2\sqrt{\left( 1-{{x}^{2}} \right) } \right) \right)$	$$\begin{bmatrix} 0.1 & 0 \\ 0 & 0.1 \end{bmatrix}$$
	Points	4002	4000

**Table 6:** Parameters for generating Artil.

Datasets	Parameters	Cluster1	Cluster2	Cluster3
Artil	Attribute1	${{x}_{1}}\in [ 10,20 ]$	${{x}_{2}}\in [ 40,120 ]$	${{x}_{3}}\in [ 20,120 ]$
	Attribute2	${{y}_{1}}\in [ 0,100 ]$	${{y}_{2}}\in [ 60,80 ]$	${{y}_{3}}\in [ 30,40 ]$
	Points	3600	3600	3600

The missing values in the real-world datasets are filled with the mean of values of all samples on the feature with missing values. All the data are normalized using the min-max method as shown in Eq. (14) to eliminate differences across different features.

14 $$\begin{aligned} {{x}_{ij}}=\frac{{{x}_{ij}}-\min \left( {{x}_{\centerdot j}} \right) }{\max \left( {{x}_{\centerdot j}} \right) -\min \left( {{x}_{\centerdot j}} \right) } \end{aligned}$$

where, ${{x}_{ij}}$ represents the value of the *j*-th feature for the *i*-th sample, while $\max \left( {{x}_{\centerdot j}} \right)$ and $\min \left( {{x}_{\centerdot j}} \right)$ represent the maximum and minimum values of all points on the *j*-th feature, respectively.

### Experiments on synthetic datasets

**Fig. 2:**
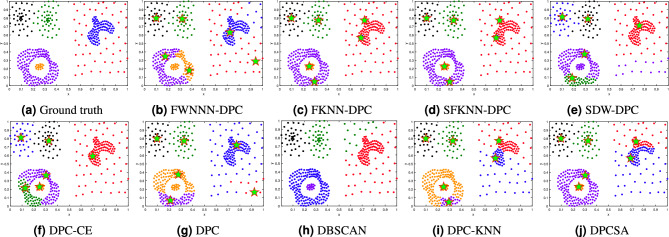
The clustering results of algorithms on Compound dataset.

**Fig. 3:**
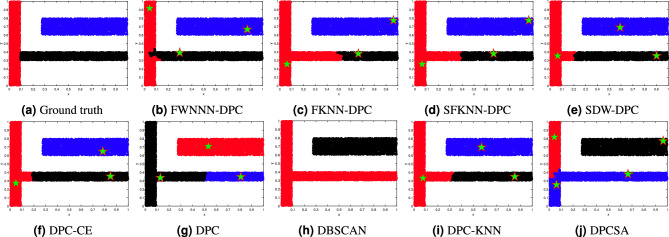
The clustering results of algorithms on Artil dataset.

**Fig. 4:**
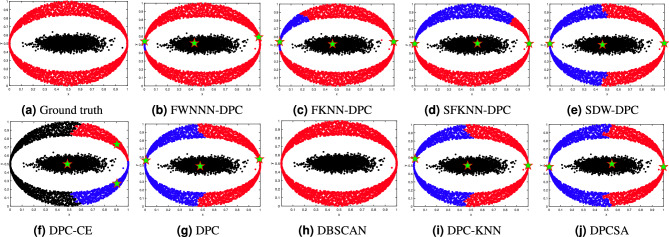
The clustering results of algorithms on Ellipse dataset.

**Fig. 5:**
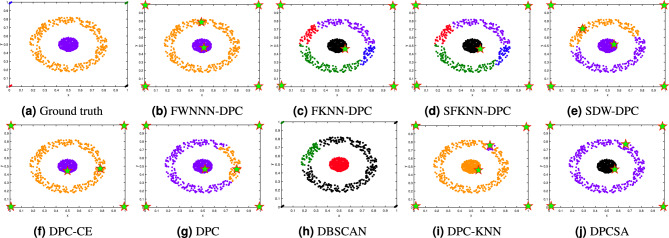
The clustering results of algorithms on Target dataset.

**Fig. 6:**
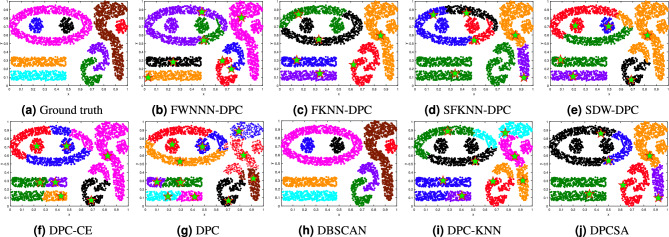
The clustering results of algorithms on Complex9 dataset.

**Fig. 7:**
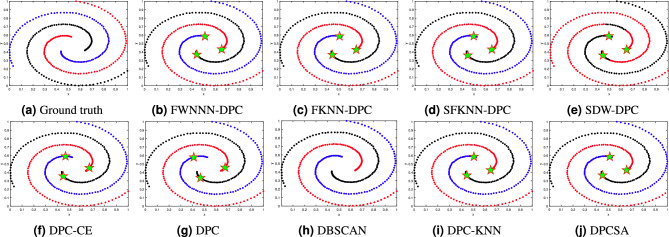
The clustering results of algorithms on Pathbased2 dataset.

This section tests the performance of the proposed FWNNN-DPC algorithm on synthetic datasets. The counterpart algorithms include FKNN-DPC, DPCSA, SFKNN-DPC, SDW-DPC, DPC-KNN, DPC-CE, DPC, K-means and DBSCAN. Figures 2, 3, 4, 5, 6 and 7 depict the clustering results of these nine algorithms, excluding K-means, on the Compound, Artil, Ellipse, Target, Complex9 and Pathbased2 datasets, respectively. To clearly show the clustering detected by FWNNN-DPC and the counterparts, the ground truth of these six synthetic datasets are also shown in Figs. 2, 3, 4, 5, 6 and 7. Additionally, Table 7 presents the performance of all algorithms on all 12 synthetic datasets from Table 3 in terms of ACC, AMI and ARI. The bold fonts indicate the best results. The *F/P* represents the number of cluster centers found by algorithms and the number of clusters covered by these detected cluster centers. The *Par* represents the parameter values of the algorithms’, and “-” indicates that the corresponding value is not available.

The Compound dataset is a very challenging dataset for clustering algorithms. The results depicted in Fig. 2 demonstrate that nearly all algorithms cannot identify the sparse back ground cluster of swallow shape. But our FWNNN-DPC can detect the swallow shape cluster from its sparse back ground cluster except for seven points from the sparse background cluster which are grouped into swallow shape cluster. However, our FWNNN-DPC cannot correctly detect the two clusters located in the bottom-left corner, due to its erroneously detect two cluster centers from the outer cluster of these two bottom-left clusters while not detecting the cluster center of the inner one. Our previously proposed FKNN-DPC and SFKNN-DPC, and the compared DPCSA and DBSCAN can detect these two clusters at the bottom-left corner. The two clusters located in upper-left corner can be detected by all algorithms in comparison.

The Artil dataset consists of three rectangular clusters, with one vertical rectangle cluster and two horizontal rectangle clusters, where one of horizontal rectangle cluster is attached to the vertical one, which leads to challenging for clustering algorithms. The results displayed in Fig. 3 demonstrate that our FWNNN-DPC performs best among nine clustering algorithms in comparison. It can detect all the clusters, though several points within two clusters attached to each other are grouped erroneously. Among the nine algorithms in comparison, only the FWNNN-DPC algorithm is able to separate the vertically rectangular cluster from the adjacent horizontal rectangle clusters relatively well except for the minor errors at the junction. FKNN-DPC, SFKNN-DPC, SDW-DPC, DPC-CE , DPC-KNN, and DPC algorithms perform similarly. They cannot correctly detect the two rectangular clusters attached to each other, though FKNN-DPC, SFKNN-DPC, SDW-DPC , DPC-KNN and DPC-CE can detect the correct cluster centers while DPC cannot. DPCSA cannot detect the correct number of clusters, nor the cluster centers and the clusters in this dataset. DBSCAN grouped two clusters attached to each other as the same one.

The Ellipse dataset is created by us utilizing the parameters displayed in Table 5. It consists of two clusters: an ellipse ring-shaped cluster encircling a smaller ellipse cluster. The results depicted in Fig. 4 demonstrate that among the nine clustering algorithms in comparison, only DBSCAN algorithm is able to detect the two clusters embedded in Ellipse dataset. Then is our FWNNN-DPC algorithm which detect three cluster centers with two from the outer ellipse ring-shaped cluster and one from the inner smaller ellipse cluster, resulting in the detection of three clusters from this dataset by partitioning the outer ellipse ring-shaped cluster into two unbalanced clusters with one containing very small number of points. The third and fourth rank is our previously proposed FKNN-DPC and SFKNN-DPC, which also detect three cluster centers from this dataset with two from the outer ellipse ring-shaped cluster and one from the inner smaller ellipse cluster, resulting in the detection of three clusters among which two from partitioning the outer one into two imbalanced clusters. DPC and other three DPC variations including SDW-DPC, DPC-KNN and DPCSA in comparison erroneously partition the ellipse ring-shaped cluster into two clusters, due to the detection of two cluster centers from this cluster. DPC-CE detects three clusters from this dataset, dividing the ellipse ring-shaped cluster into three clusters, with the largest one among which being mistakenly assigned to the inner ellipse cluster.

It can be noticed from the results depicted in Fig. 4 that FWNNN-DPC, DPC and its variants in comparison simultaneously detect three cluster centers from this Ellipse dataset, with two from the outer ellipse ring-shaped cluster and one from the inner smaller ellipse cluster, resulting in the erroneous in the detection of the clusters within this dataset. Moreover, the results depicted in Fig. 4 also demonstrate that the amounts of points erroneously grouped into another clusters are different within the clustering results of our FWNNN-DPC and the original DPC and the other DPC variants in comparison, such as FKNN-DPC, SFKNN-DPC, SDW-DPC, and DPCSA, which somewhat demonstrate the capability of the assignment strategies proposed by each algorithm and the validity of the local density definition in each algorithm. Hence, it can be conclude that the contributions of our FWNNN-DPC are somewhat powerful in detecting correct clusters of a dataset as far as possible, resulting in its superiority to DPC and its variants.

Furthermore, it can be noticed from the results of our FWNNN-DPC and DPC and the other DPC variants in comparison depicted in Fig. 4 that the two cluster centers erroneously detected from the outer ellipse ring-shaped cluster by these algorithms locate in the very similar places, which indicate we need further investigate the method for detecting the density peaks within a dataset, even the definition of the local density of a point, so as to guarantee the correct number of density peaks are to be found, and the correct cluster centers are to be detected.

The Target dataset consists of six clusters: one ring-shaped cluster encircling one Gaussian distribution cluster, and four very small clusters at four corners. The clustering results depicted in Fig. 5 show that among nine algorithms in comparison, only FWNNN-DPC, DPC-CE and DPCSA algorithms are able to detect these six clusters within the Target dataset.

Our previously proposed FKNN-DPC and SFKNN-DPC algorithm cannot detect the cluster center of the ring-shaped cluster within this dataset, resulting in the partition of this cluster into four clusters which are, respectively, grouped into those four corner clusters.

Although DPC, SDW-DPC and DPC-KNN algorithms can successfully detect six cluster centers, they failed in detecting the ring-shaped cluster of this dataset. On the contrary, they partition the ring-shaped cluster into two clusters, and the cluster containing majority points of ring-shaped cluster are grouped together with the Gaussian distribution cluster circled by the ring-shaped cluster, particularly the DPC-KNN algorithm.

DBSCAN algorithm detects three clusters from the Target dataset by partitioning the ring-shaped cluster into two clusters, and groups three corner clusters of this dataset together with the cluster comprising majority points of the ring-shaped cluster while the other one corner cluster is grouped together with the cluster comprising the minority points of the ring-shaped cluster.

The Complex9 dataset is one of the challenging datasets for testing the performance of a clustering algorithm. The results displayed in Fig. 6 demonstrate the clustering performance of our FWNNN-DPC and other eight counterpart clustering algorithms on the Complex9 dataset.

The clustering results in Fig. 6 show that among nine algorithms, the primary density-based clustering algorithm DBSCAN performs best by correctly identifying all clusters within Complex9 dataset, followed by our proposed algorithm FWNNN-DPC, which detects seven cluster centers from six clusters while missing the cluster centers of three spherical small clusters, resulting in the elliptical cluster in the upper left corner being partitioned into two clusters while the three spherical small clusters cannot be detected, including two circled by the elliptical cluster while being grouped into the clusters encompassing them.

The SDW-DPC identifies six correct cluster centers while missing three ones, particularly the one of the elliptical cluster, resulting in its being classified into three clusters. Moreover, the right side spherical small cluster is grouped into the reverse-question-mark-shaped cluster which encompasses it. The right side upper u-shaped cluster is partitioned into two clusters, due to its cluster center not being detected.

The DPC-CE detects seven cluster centers from six clusters while missing the cluster center of the elliptical cluster and that of the right side spherical small cluster and that of the upper u-shaped cluster, resulting in the elliptical cluster being partitioned into three clusters, and the right side spherical small cluster is grouped into the reverse-question-mark-shaped cluster which encompasses it, and the right side upper u-shaped cluster is partitioned into two clusters. Furthermore, DPC-CE erroneously detects two cluster centers from the upper rectangle cluster in the lower-left corner, resulting in its being partitioned into two clusters.

FKNN-DPC identifies six cluster centers from five clusters while missing detecting cluster centers of four clusters including the three spherical small clusters and the u-shaped cluster above the other. It detects two cluster centers from the elliptical cluster, resulting in its being grouped into two clusters, and the two spherical small clusters encompassed by it are grouped into these two clusters. The other spherical small cluster is grouped into the reverse-question-mark-shaped cluster which encompasses it. The u-shaped cluster without detecting cluster center is assigned to the other u-shaped cluster with being detected cluster center.

The DPCSA detects six cluster centers from four clusters while missing the ones of the other five clusters. It incorrectly splits the elliptical cluster into two clusters due to its detecting two cluster centers from it, so does to the reverse-question-mark-shaped cluster. It groups clusters without being detected cluster centers into the near ones which have been detected cluster centers.

The DPC-KNN algorithm identifies eight cluster centers from five clusters while missing the cluster center of the three spherical small clusters and that of the upper u-shaped cluster. It detects two clusters from the elliptical cluster due to its detecting two cluster centers from it. Additionally, it splits the reverse-question-mark-shaped cluster into three ones due to its detecting three cluster centers from it. It erroneously groups the three spherical small clusters into the ones near them. The worst is that it assign half of the points from the below rectangle cluster into the upper one, though it detects the correct cluster centers of these two rectangle clusters. Furthermore, it splits the upper u-shaped cluster into two and grouped them together with the near two clusters, respectively.

DPC detects 11 cluster centers from seven cluster while missing the ones of the upper u-shaped cluster and the spherical cluster encompassed by the reverse-question-mark-shaped cluster. It detects too many clusters from the Complex9 dataset, particularly partitions each of the two rectangle clusters into two, and partitions the reverse-question-mark-shaped cluster into three, even partitions the upper u-shaped cluster into three. All in all the performance of DPC on this dataset is very bad.

The SFKNN-DPC performs worst on this dataset. It detects six cluster centers from three clusters, including detecting three from the elliptical cluster, and two from the reverse-question-mark-shaped cluster. It fails to detect the cluster centers of the three spherical clusters and of two u-shaped clusters, even of the upper rectangle cluster.

The Pathbased2 dataset comprises three distinct spiral clusters characterized by diminishing densities of data points as one moves from the center towards the periphery. The results displayed in Fig. 7 demonstrate the clustering performance of our FWNNN-DPC and other eight clustering algorithms on the Pathbased2 dataset. It can bee seen that SDW-DPC performs worst among the nine algorithms in comparison. The remaining eight algorithms have achieved the satisfactory clustering results for the Pathbased2 dataset, finding each cluster within it.

The clustering outcomes depicted in Figs. 2, 3, 4, 5, 6 and 7 demonstrate the superiority of our FWNNN-DPC over the counterparts, which can be largely attributed to its intrinsic stability. Specifically, the adaptive natural nearest neighbors of a point, found by Algorithm 1, ensure the stability of its local density estimation. This stability further propagates to the subsequent assignment strategies, which are entirely guided by the natural nearest neighbor relationships. These properties together lead to the stable clustering outcome of FWNNN-DPC. The whole procedure is parameter-free. However, everything has two sides. This parameter-free property of FWNNN-DPC results in its inability in some cases. For examples, FWNNN-DPC fails to detect the centers of three small spherical clusters in the Complex9 dataset and one such cluster in the Compound dataset, while detecting redundant cluster centers from the outer elliptical ring-shaped cluster of the Ellipse dataset and the outer ring cluster of the Compound dataset. These observations indicate that FWNNN-DPC exhibits a degree of sensitivity to data geometry and relative spatial configuration, especially in scenarios involving closely nested cluster structures.

The experimental results depicted in Table 7 demonstrate that the proposed FWNNN-DPC algorithm has achieved satisfactory performance on most of the synthetic datasets. Specifically, on the Compound dataset, the FWNNN-DPC ranked the second after DBSCAN in terms of ACC. On the A3 dataset, the FWNNN-DPC ranked the third in terms of ARI metric, after DPC-KNN and SDW-DPC. Moreover, the FWNNN-DPC tied with DPC in the second place in terms of ACC, trailing DPC-KNN, and was also tied for second with both the DPC and SDW-DPC algorithms in terms of AMI, again following DPC-KNN.

**Table 7:** Performances of different clustering algorithms on different synthetic datasets.

Algorithm	Compound					A3					Target				
	ACC	AMI	ARI	F/P	Par	ACC	AMI	ARI	F/P	Par	ACC	AMI	ARI	F/P	Par
FWNNN-DPC	0.860	0.817	0.742	6/5	–	0.989	0.986	0.978	50/50	–	**1**	**1**	**1**	**6/6**	–
FKNN-DPC	0.857	**0.846**	0.844	6/5	5	0.987	0.985	0.975	50/50	10	0.691	0.531	0.671	5/5	7
SFKNN-DPC	0.857	**0.846**	0.844	6/5	5	0.987	0.985	0.974	50/50	11	0.691	0.531	0.671	5/5	7
SDW-DPC	0.712	0.697	0.576	5/4	2	0.990	0.986	0.979	50/50	1.25	0.742	0.365	0.255	6/6	2
DPC-KNN	0.747	0.734	0.640	6/5	1.5	**0.991**	**0.988**	**0.983**	50/50	1	0.549	0.143	0.061	6/6	1
DPC-CE	0.734	0.828	0.643	6/5	–	0.743	0.591	0.469	3/2	–	**1**	**1**	**1**	**6/6**	–
DPCSA	0.840	0.839	0.828	6/5	–	0.082	0.346	0.112	4/4	–	**1**	**1**	**1**	**6/6**	–
DPC	0.852	0.769	0.791	5/5	2	0.989	0.986	0.977	50/50	1.25	0.529	0.125	0.058	5/5	2
K-means	0.601	0.657	0.523	–	6	0.795	0.927	0.809	–	50	0.556	0.459	0.549	–	6
DBSCAN	**0.867**	0.804	**0.850**	–	0.05/6	0.907	0.915	0.899	–	0.01/6	0.907	0.762	0.852	–	0.04/6

**Algorithm**	Artil					Aggregation					Complex9				
	ACC	AMI	ARI	F/P	Par	ACC	AMI	ARI	F/P	Par	ACC	AMI	ARI	F/P	Par
FWNNN-DPC	**0.985**	**0.935**	**0.956**	3/3	–	0.992	0.978	0.986	7/7	–	0.886	0.862	0.806	7/6	–
FKNN-DPC	0.844	0.747	0.653	3/3	12	**0.999**	0.995	0.997	7/7	8	0.749	0.806	0.711	6/5	13
SFKNN-DPC	0.892	0.790	0.734	3/3	8	**0.999**	0.995	0.997	7/7	8	0.482	0.621	0.401	6/3	6
SDW-DPC	0.959	0.884	0.885	3/3	6	0.996	0.987	0.992	7/7	2	0.718	0.667	0.536	6/6	2
DPC-KNN	0.915	0.816	0.780	3/3	10	0.938	0.920	0.917	7/7	1	0.554	0.657	0.420	8/5	1
DPC-CE	0.966	0.897	0.902	3/3	–	**0.999**	**0.996**	**0.998**	7/7	–	0.619	0.644	0.486	7/6	–
DPCSA	0.870	0.764	0.697	4/3	–	0.973	0.954	0.958	7/7	–	0.569	0.713	0.558	6/4	–
DPC	0.849	0.751	0.661	3/2	10	0.997	0.992	0.996	7/7	4	0.551	0.656	0.459	11/7	2
K-means	0.650	0.486	0.403	–	3	0.747	0.789	0.695	–	7	0.476	0.593	0.372	–	9
DBSCAN	0.667	0.761	0.571	–	0.05/6	0.996	0.987	0.992	–	0.05/8	**1**	**1**	**1**	–	0.025/6

Algorithm	Zelnik3					Ellipse					Zelnik1				
	ACC	AMI	ARI	F/P	Par	ACC	AMI	ARI	F/P	Par	ACC	AMI	ARI	F/P	Par
FWNNN-DPC	**1**	**1**	**1**	3/3	–	0.974	0.933	0.951	3/2	–	0.796	0.606	0.630	2/2	–
FKNN-DPC	**1**	**1**	**1**	3/3	8	0.909	0.861	0.851	3/2	20	0.789	0.605	0.582	5/2	20
SFKNN-DPC	**1**	**1**	**1**	3/3	8	0.814	0.822	0.766	3/2	7	0.763	0.568	0.480	3/2	16
SDW-DPC	0.940	0.800	0.818	3/3	1.5	0.759	0.815	0.750	3/2	2	0.753	0.604	0.576	3/2	0.25
DPC-KNN	0.797	0.583	0.488	3/3	2	0.759	0.815	0.750	3/2	0.5	0.579	0.256	0.188	3/2	1
DPC-CE	**1**	**1**	**1**	3/3	–	0.623	0.284	0.166	3/2	–	**1**	**1**	**1**	**3/3**	–
DPCSA	**1**	**1**	**1**	3/3	–	0.755	0.815	0.750	3/2	–	**1**	**1**	**1**	**3/3**	–
DPC	0.797	0.583	0.488	3/3	2	0.757	0.815	0.750	3/2	2	0.756	0.604	0.580	3/2	0.25
K-means	0.800	0.586	0.494	–	3	0.531	0.003	0.004	–	2	0.458	0.156	0.053	–	3
DBSCAN	**1**	**1**	**1**	–	0.09/5	**0.999**	**0.999**	**0.998**	–	0.05/6	**1**	**1**	**1**	–	0.04/5

Algorithm	Cdataset1					DIM512					Pathbased2				
	ACC	AMI	ARI	F/P	Par	ACC	AMI	ARI	F/P	Par	ACC	AMI	ARI	F/P	Par
FWNNN-DPC	0.932	0.779	0.815	4/4	–	**1**	**1**	**1**	16/16	–	**1**	**1**	**1**	3/3	–
FKNN-DPC	0.930	0.769	0.814	4/4	23	**1**	**1**	**1**	16/16	8	**1**	**1**	**1**	3/3	6
SFKNN-DPC	0.942	0.789	0.842	4/4	8	**1**	**1**	**1**	16/16	8	**1**	**1**	**1**	3/3	8
SDW-DPC	0.867	0.618	0.647	4/4	3	**1**	**1**	**1**	16/16	2	0.606	0.367	0.396	3/3	0.25
DPC-KNN	0.930	0.770	0.812	4/4	3	**1**	**1**	**1**	16/16	2	**1**	**1**	**1**	3/3	2
DPC-CE	0.940	0.785	0.835	4/4	–	**1**	**1**	**1**	16/16	–	**1**	**1**	**1**	3/3	–
DPCSA	0.917	0.740	0.788	4/4	–	**1**	**1**	**1**	16/16	–	**1**	**1**	**1**	3/3	–
DPC	0.815	0.535	0.547	4/4	1.5	**1**	**1**	**1**	16/16	2	**1**	**1**	**1**	3/3	2
K-means	**0.954**	**0.831**	**0.882**	–	4	0.765	0.867	0.751	–	16	0.343	0.006	0.006	–	3
DBSCAN	0.908	0.773	0.875	–	0.05/6	**1**	**1**	**1**	–	0.3/7	**1**	**1**	**1**	–	0.1/5

Significant values are in bold.

On the Target dataset, the FWNNN-DPC, DPC-CE and DPCSA algorithms achieved perfect clustering results and obtained the highest values in three metrics. On the Artil dataset, the FWNNN-DPC algorithm obtained the highest values in terms of three metrics of ACC, AMI and ARI, significantly outperforming the other nine algorithms in comparison. On the Aggregation dataset, the results of the FWNNN-DPC algorithm were not good in ACC, AMI and ARI metrics, but they were very close. On the Complex9 dataset, the FWNNN-DPC algorithm’s performance ranked the second following that of DBSCAN algorithm’s, and superior to that of the other algorithms’ in comparison. On the Zelnik3 dataset, only SDW-DPC, DPC-KNN, DPC and K-means algorithm performed poorly, while the other algorithms successfully identified all clusters. On the Ellipse dataset, the FWNNN-DPC algorithm ranked second following DBSCAN algorithm in terms of ACC, AMI and ARI. On the Zelnik1 dataset, the FWNNN-DPC algorithm ranked the second following DBSCAN, DPC-CE and DPCSA algorithms, due to DBSCAN, DPC-CE and DPCSA correctly detecting all clusters within this dataset. On the Cdataset1 dataset, the FWNNN-DPC algorithm was slightly inferior to K-means, DPC-CE and SFKNN-DPC algorithms in terms of ACC, AMI and ARI metrics. On the DIM512 dataset, only K-means algorithm performed poorly, while the other algorithms successfully identified all clusters. On the Pathbased2 dataset, only SDW-DPC and K-means algorithms performed poorly, while the other algorithms successfully identified all clusters.

It should be noted that we compared the performance of our FWNNN-DPC to the best clustering results of the other nine clustering algorithms in comparison. So, we can conclude that our FWNNN-DPC performs well, particularly it is superior to other eight clustering algorithms in comparison in detecting the clusters within the very challenging synthetic datasets, which can be seen from the clustering results depicted in Figs. 2, 3, 4, 5, 6 and 7.

### Experiments on real-world datasets

To further test the performance of the proposed FWNNN-DPC algorithm in detecting the clusters within a dataset from real-world scenarios, this study choose 14 popular real-world datasets from UCI machine learning repository^41^ to carry out experiments. There are large differences in dimensionalities, number of samples and number of clusters between these real-world datasets from UCI machine learning repository. Therefore, we can test the performance of our FWNNN-DPC and the counterparts in detecting the clusters within a real-world dataset. The details of these datasets are shown in Table 3. We will compare the performance of our FWNNN-DPC to that of DPC and its variants including FKNN-DPC, DPCSA, SFKNN-DPC, SDW-DPC, DPC-KNN and DPC-CE, and that of the classic very famous clustering algorithms such as K-means and DBSCAN. The evaluation metrics comprise ACC, AMI and ARI.

Table 8 displayed the clustering results of each algorithm on the real-world datasets from UCI machine learning repository. The bold fonts indicates the best results. The *F/P* represents the number of cluster centers found by algorithms and the number of clusters covered by the detected cluster centers. The *Par* represents the parameter values of algorithms, and “-” indicates that there is not corresponding value.

**Table 8:** Performance comparison of different clustering algorithms on different real-world datasets.

Algorithm	Libras					Ecoli					Yeast				
	ACC	AMI	ARI	F/P	Par	ACC	AMI	ARI	F/P	Par	ACC	AMI	ARI	F/P	Par
FWNNN-DPC	**0.497**	**0.542**	**0.353**	15/11	–	**0.813**	**0.643**	**0.747**	5/5	–	**0.480**	**0.261**	**0.220**	8/5	–
FKNN-DPC	0.436	0.508	0.308	13/9	9	0.774	0.596	0.730	6/5	6	0.350	0.115	0.079	7/5	4
SFKNN-DPC	0.464	0.529	0.344	12/10	9	0.777	0.600	0.723	6/5	6	0.317	0.027	0.010	7/6	8
SDW-DPC	0.339	0.405	0.195	13/9	0.5	0.646	0.395	0.440	7/5	1	0.320	0.126	0.012	7/4	0.25
DPC-KNN	0.456	0.520	0.328	12/9	2	0.649	0.389	0.430	6/5	1	0.320	0.128	0.012	7/6	0.2
DPC-CE	0.475	0.533	0.322	15/12	–	0.792	0.611	0.733	5/5	–	0.397	0.132	0.116	3/3	–
DPCSA	0.422	0.486	0.264	14/10	–	0.699	0.419	0.479	7/5	–	0.318	0.121	0.011	8/5	–
DPC	0.361	0.390	0.214	9/7	0.5	0.646	0.392	0.435	8/6	2	0.318	0.121	0.011	8/5	0.0215
K-means	0.443	0.519	0.304	–	15	0.641	0.574	0.525	–	6	0.403	0.248	0.159	–	8
DBSCAN	0.350	0.408	0.154	–	0.96/5	0.560	0.392	0.473	–	0.1/3	0.345	0.112	0.060	–	0.1/3

Algorithm	Glass					Balance scale					Waveform				
	ACC	AMI	ARI	F/P	Par	ACC	AMI	ARI	F/P	Par	ACC	AMI	ARI	F/P	Par
FWNNN-DPC	**0.514**	**0.328**	**0.282**	5/4	–	0.456	**0.419**	**0.339**	**4/4**	–	0.565	**0.374**	0.265	3/3	–
FKNN-DPC	0.505	0.262	0.185	5/4	3	0.416	0.215	0.190	3/2	25	**0.703**	0.324	**0.350**	3/3	5
SFKNN-DPC	0.449	0.215	0.133	5/3	3	0.416	0.248	0.236	3/2	25	0.656	0.309	0.301	3/3	5
SDW-DPC	0.481	0.224	0.184	4/4	0.5	0.264	0.234	0.078	7/2	1	0.521	0.358	0.281	3/3	1.5
DPC-KNN	0.453	0.165	0.177	6/5	2	0.320	0.226	0.077	7/3	50	0.670	0.329	0.291	3/2	1
DPC-CE	0.477	0.225	0.177	5/4	–	**0.467**	0.015	0.005	9/2	–	0.515	0.365	0.280	3/3	–
DPCSA	0.453	0.165	0.177	6/5	–	0.061	0.108	0.023	81/3	–	0.628	0.260	0.233	3/3	–
DPC	0.453	0.182	0.135	6/4	0.5	0.368	0.328	0.154	7/3	5	0.586	0.318	0.268	3/3	0.5
K-means	0.438	0.288	0.177	–	6	0.296	0.102	0.085	–	3	0.501	0.364	0.254	–	3
DBSCAN	0.360	0.213	0.110	–	0.1/3	0.239	**0.419**	0.066	0.5/1	–	–	–	–	–	–

Algorithm	Waveform(noise)					Pima-indians-diabetes					Iris				
	ACC	AMI	ARI	F/P	Par	ACC	AMI	ARI	F/P	Par	ACC	AMI	ARI	F/P	Par
FWNNN-DPC	0.503	0.362	0.248	3/3	–	0.622	0.041	0.059	2/1	–	0.953	0.838	0.867	3/3	–
FKNN-DPC	0.648	0.247	0.253	3/3	5	0.648	0.001	0.013	2/2	6	**0.973**	**0.912**	**0.922**	3/3	7
SFKNN-DPC	**0.693**	0.278	**0.302**	3/3	5	0.648	0.001	0.013	2/2	6	0.967	0.883	0.904	3/3	7
SDW-DPC	0.525	0.160	0.097	3/3	2	0.647	0.009	0.040	2/1	3	0.967	0.883	0.904	3/3	1.5
DPC-KNN	0.544	0.193	0.181	3/3	0.2	0.650	0.002	0.014	2/2	3	0.960	0.861	0.886	3/3	2
DPC-CE	0.545	0.197	0.157	3/3	–	0.664	0.015	0.025	2/2	–	0.853	0.728	0.663	3/3	–
DPCSA	0.524	0.152	0.135	3/3	–	0.652	0.001	0.002	2/2	–	0.967	0.883	0.904	3/3	–
DPC	0.535	0.184	0.164	3/3	0.3	0.650	0.034	0.078	2/1	4	0.887	0.767	0.720	3/3	2
K-means	0.512	**0.364**	0.252	–	3	**0.668**	**0.050**	**0.102**	–	2	0.825	0.692	0.660	–	3
DBSCAN	–	–	–	–	–	0.540	0.017	0.035	–	0.15/6	0.893	0.775	0.732	–	0.14/9

Algorithm	Wdbc					Thyroid					Dermatology				
	ACC	AMI	ARI	F/P	Par	ACC	AMI	ARI	F/P	Par	ACC	AMI	ARI	F/P	Par
FWNNN-DPC	0.933	0.644	0.750	2/2	–	**0.902**	**0.577**	**0.705**	4/3	–	0.719	0.775	**0.760**	10/5	–
FKNN-DPC	0.944	0.679	0.786	2/2	7	0.874	0.474	0.590	3/3	10	0.768	**0.847**	0.718	6/5	7
SFKNN-DPC	**0.958**	**0.735**	**0.837**	2/2	5	0.833	0.334	0.440	3/3	10	0.784	0.731	0.666	7/6	4
SDW-DPC	0.854	0.422	0.491	2/2	6	0.823	0.309	0.410	3/3	8	**0.836**	0.786	0.753	6/5	0.2
DPC-KNN	0.863	0.455	0.518	2/2	3	0.749	0.195	0.262	3/2	3	0.792	0.735	0.691	5/5	4
DPC-CE	0.896	0.508	0.627	2/1	–	0.740	0.123	0.183	3/2	–	0.336	0.165	0.059	6/5	–
DPCSA	0.814	0.336	0.377	2/2	–	0.795	0.231	0.319	3/3	–	0.712	0.745	0.606	6/5	–
DPC	0.613	0.009	0.011	2/1	9	0.795	0.231	0.319	3/3	0.2	0.697	0.588	0.490	4/4	2
K-means	0.928	0.611	0.730	–	2	0.884	0.508	0.617	–	3	0.691	0.786	0.654	–	6
DBSCAN	0.870	0.419	0.542	–	0.27/7	0.795	0.388	0.495	–	0.2/2	0.787	0.709	0.727	–	0.7/3

Algorithm	Spambase					Letter									
	ACC	AMI	ARI	F/P	Par	ACC	AMI	ARI	F/P	Par					
FWNNN-DPC	0.625	0.072	0.042	3/2	–	**0.321**	0.402	0.081	30/20	–					
FKNN-DPC	0.624	0.063	0.021	2/2	5	0.251	0.312	0.063	29/19	5					
SFKNN-DPC	0.601	0.018	0.004	2/2	5	0.195	0.256	0.049	30/17	5					
SDW-DPC	0.602	0.016	0.002	3/2	0.2	0.251	0.376	0.054	30/20	0.2					
DPC-KNN	0.581	0.046	0.015	2/1	2	0.225	0.254	0.065	28/18	2					
DPC-CE	0.606	0.006	0.001	2/2	–	0.187	0.209	0.054	27/16	–					
DPCSA	0.603	0.014	0.002	2/1	–	0.245	**0.443**	0.040	28/17	–					
DPC	0.602	0.016	0.003	2/2	2	0.183	0.194	0.063	28/17	8					
K-means	**0.704**	0.010	0.005	–	2	0.258	0.347	**0.132**	–	26					
DBSCAN	0.533	**0.141**	**0.062**	–	0.2/2	0.239	0.419	0.066	–	0.2/8					

Significant values are in bold.

The experimental results displayed in Table 8 demonstrate that, in most of the real-world datasets considered, the clustering performance of the FWNNN-DPC is comparable to or even better than that of other algorithms. Specifically, on Libras, Ecoli, Yeast, Glass and Thyroid datasets, our FWNNN-DPC performs best in terms of ACC, AMI and ARI metrics. On Balance scale dataset, FWNNN-DPC obtains the highest AMI and ARI values, and the second-highest ACC value following DPC-CE.

On Waveform dataset, FWNNN-DPC obtains the highest AMI value, while its ACC and ARI values are not good. However, our previously proposed FKNN-DPC obtains the best ACC and ARI values on this dataset. On Waveform (noise) dataset, FWNNN-DPC obtains the second-highest AMI value following K-means. Our previously proposed SFKNN-DPC performs best on this dataset in terms of ACC and ARI. There are no related results of DBSCAN on these two waveform datasets, due to the difficulty in tuning the best parameters for DBSCAN on these two datasets.

On Pima-indians-diabetes dataset, K-means obtains the highest values in terms of all three metrics. Our FWNNN-DPC achieves the second-highest AMI value and the third-highest ARI value on Pima-indians-diabetes dataset.

On Iris dataset, the performance of our previously proposed FKNN-DPC outperforms the counterparts in terms of ACC, AMI and ARI. Our FWNNN-DPC only outperforms original DPC and its variant DPC-CE and the typical density based clustering algorithm DBSCAN and the very popular partition-based clustering algorithm K-means, but inferior to other DPC variants in comparison. Although our FWNNN-DPC was defeated by our previously proposed FKNN-DPC and SFKNN-DPC on Iris dataset, the results of FKNN-DPC and SFKNN-DPC were obtained by tuning the parameter K while our FWNNN-DPC do not need tuning any parameter. Similar causes exist in SDW-DPC and DPC-KNN.

On Wdbc dataset, our previously proposed SFKNN-DPC is superior to other counterparts, our FWNNN-DPC ranks the third in terms of ACC, AMI and ARI, following our previously proposed FKNN-DPC and SFKNN-DPC algorithm.

On Dermatology dataset, our FWNNN-DPC obtains the highest ARI value, while its ACC and AMI values are not the best. However, our previously proposed FKNN-DPC and SDW-DPC are, respectively, superior to other counterparts in terms of AMI and ACC.

On Spambase dataset, the FWNNN-DPC obtains the second-highest ACC value following K-means, and the second-highest AMI and ARI values following DBSCAN. On Letter dataset, FWNNN-DPC defeated all counterparts in terms of ACC, and ranked the third in terms of AMI and the second in terms of ARI. The DPCSA is superior to the counterparts in terms of AMI while K -means defeats the counterparts in terms of ARI.

Summarizing the aforementioned analyses of the experimental results of FWNNN-DPC and counterparts on 14 real-wold datasets from UCI machine learning repository, we can conclude that FWNNN-DPC outperforms counterparts in most cases but not always. Though it does not need manually setting parameters. Hence, it is necessary to test whether FWNNN-DPC is statistically significantly different to the counterparts.

### Statistical significant test

In this subsection, a statistical significance test will be conducted to verify whether there is significant difference between our FWNNN-DPC and the peers including FKNN-DPC, DPCSA, SFKNN-DPC, SDW-DPC, DPC-KNN, DPC-CE, DPC, K-means and DBSCAN algorithms. The Friedman’s test will be conducted first to determine whether there is significant differences among these 10 algorithms. If the significant differences are found, the Nemenyi’s test will be conducted as the *post-hoc* test to examine the statistical significance differences between pairwise algorithms. The Friedman’s test will be conducted at $\alpha =0.05$ utilizing the clustering results of these 10 clustering algorithms in terms of ACC, AMI and ARI on both synthetic and real-world datasets.

The results of the Friedman’s test are as follows: For ACC, ${{\chi }^{2}}$=39.36, *df*=9, *p*=9.9122e-06; For AMI, ${{\chi }^{2}}$=38.41, *df*=9, *p*=1.46924e-05; For ARI, ${{\chi }^{2}}$=42.47, *df*=9, *p*=2.69714e-06. The *p* values of Friedman’s test for ACC, AMI, and ARI are simultaneously far less than $\alpha$, so we conclude that there are strong significant differences among these 10 clustering algorithms.

Therefore, we need further conduct the Nemenyi’s test to detect whether there is statistically significant differences between pairwise algorithms. If the mean rank difference between pairwise algorithms is less than threshold *CD*, then the null hypothesis that there is no significant difference between the pairwise algorithms will be accepted at the confidence level of $1-\alpha$; otherwise, the null hypothesis will be rejected, and accept the alternative hypothesis, that is, there is significant difference between the pairwise algorithms. The threshold *CD* is defined in Eq. (15).

15 $$\begin{aligned} CD={{q}_{\alpha }}\sqrt{\frac{M\left( M+1 \right) }{6N}} \end{aligned}$$

where, *M* represents the number of algorithms, and *N* the number of datasets. The ${{q}_{\alpha }}$ is the threshold of Tukey distribution, and its value can be obtained by consulting statistic textbook.

**Fig. 8:**
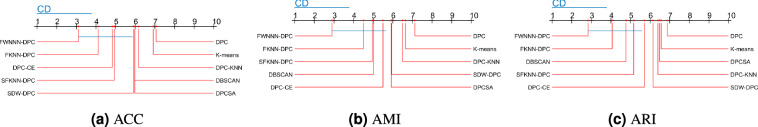
The Nemenyi’s test results of 10 clustering algorithms.

For our Nemenyi’s test, the *M*=10, *N*=24 not 26 because it is challenging to tune the best parameters for DBSCAN on Waveform and Waveform (noise) datasets, resulting in “-” for DBSCAN on these two datasets in terms of ACC, AMI and ARI. So, the ${{q}_{\alpha }}$=3.164, and the corresponding *CD*=2.765. The results of the Nemenyi’s test through ACC, AMI and ARI at confidence level of 0.95(=$1-\alpha$) are shown in Fig. 8.

The results depicted in Fig. 8 demonstrate that our FWNNN-DPC algorithm outperforms the other nine clustering algorithms in comparison no matter in terms of ACC, AMI and ARI. In addition, the Nemenyi’s test results depicted in Fig. 8a demonstrate that FWNNN-DPC is statistically significantly different from the other six algorithms except for FKNN-DPC, DPC-CE and SFKNN-DPC in terms of ACC. Moreover, the Nemenyi’s test results depicted in Fig. 8b demonstrate that our FWNNN-DPC is statistically significantly different to DPC, K-means, DPC-KNN, SDW-DPC and DPCSA algorithms while there is not statistically significant difference between it and any one of FKNN-DPC, SFKNN-DPC, DBSCAN and DPC-CE algorithms in terms of AMI. Furthermore, the Nemenyi’s test results depicted in Fig. 8c demonstrate that our FWNNN-DPC is statistically significantly different to DPC, K-means, DPCSA, DPC-KNN, SDW-DPC and DPC-CE algorithms while there is not statistically significant difference between it and any one of FKNN-DPC, DBSCAN and SFKNN-DPC algorithms in terms of ARI.

Although FWNNN-DPC is not significantly different from FKNN-DPC, DPC-CE and SFKNN-DPC algorithms in terms of ACC, it outperforms these counterparts. In addition, although FWNNN-DPC is not significantly different from FKNN-DPC, SFKNN-DPC, DBSCAN and DPC-CE in terms of AMI, it performs better than them. Moreover, although the proposed FWNNN-DPC is not significantly different from FKNN-DPC, DBSCAN and SFKNN-DPC in terms of ARI, it is superior to these counterparts.

These results further indicate that the innovations in FWNNN-DPC including the definition of the new local density of a point and the new assignment strategies for assigning non-cluster center points to the most appropriate clusters based on our new natural nearest neighbors do great contributions to enhance the clustering performance of the proposed FWNNN-DPC algorithm.

### Ablation experiments

This subsection will conduct ablation experiments to test the capability of each contribution of our FWNNN-DPC algorithm’s in enhancing its performance. There are two main contributions in FWNNN-DPC algorithm. One is the definition of the new local density of a point by introducing the new natural nearest neighbors, referred to NLD. The other is the new assignment strategies based on the new natural nearest neighbors, referred to NAS. The base algorithm is our previous proposed FKNN-DPC^21^. We conduct ablation experiments on synthetic datasets from Table 3 except for Aggregation, Zelnik3, DIM512 and Pathbased2 datasets because the performances of the majority of algorithms on these four datasets are very close to each other, or even the same. The ablation experimental results are shown in Table 9. The bold fonts mean the best results. The FKNN-DPC+NLD indicates that the local density definition of a point in FKNN-DPC utilized that of FWNNN-DPC, and FKNN-DPC+NAS indicates that the assignment strategies in FKNN-DPC utilizes that in FWNNN-DPC.

**Table 9:** The ablation experimental results to test the innovations proposed in FWNNN-DPC algorithm with FKNN-DPC algorithm as the base algorithm in terms of ACC, AMI and ARI.

Algorithm	Compound			A3			Target			Artil		
	ACC	AMI	ARI	ACC	AMI	ARI	ACC	AMI	ARI	ACC	AMI	ARI
FKNN-DPC	0.857	**0.846**	**0.844**	0.987	0.985	0.975	0.691	0.531	0.671	0.884	0.747	0.653
FKNN-DPC+NLD	0.792	0.780	0.656	0.875	0.940	0.835	**1.000**	**1.000**	**1.000**	**0.985**	**0.935**	**0.956**
FKNN-DPC+NAS	**0.862**	0.820	0.832	0.954	0.964	0.922	0.984	0.884	0.970	0.670	0.753	0.570
FWNNN-DPC	0.860	0.817	0.742	**0.989**	**0.986**	**0.978**	**1.000**	**1.000**	**1.000**	**0.985**	**0.935**	**0.956**

Algorithm	Zelnik1			Complex9			Cdataset1			Ellipse		
	ACC	AMI	ARI	ACC	AMI	ARI	ACC	AMI	ARI	ACC	AMI	ARI
FKNN-DPC	0.789	0.605	0.582	0.749	0.806	0.711	0.930	0.769	0.814	0.909	0.861	0.851
FKNN-DPC+NLD	0.796	0.606	0.630	**0.821**	**0.814**	**0.781**	**0.933**	0.773	**0.817**	**0.974**	**0.933**	**0.951**
FKNN-DPC+NAS	**0.990**	**0.949**	**0.970**	0.749	0.806	0.711	0.867	0.669	0.654	0.942	0.890	0.897
FWNNN-DPC	0.796	0.606	0.630	**0.821**	**0.814**	**0.781**	0.932	**0.779**	0.815	**0.974**	**0.933**	**0.951**

Significant values are in bold.

The ablation experimental results depicted in Table 9 demonstrate that the NLD innovation of FWNNN-DPC significantly enhanced the performance of the base algorithm FKNN-DPC on most of datasets except for Compound and A3 datasets, but these two innovations NLD and NAS together enhanced the performance of the proposed FWNNN-DPC algorithm on A3 datasets in terms of ACC, AMI and ARI metrics, and on Compound dataset in terms of ACC. Moreover, only one contribution NAS can advance the base algorithm FKNN-DPC to the highest ACC on Compound dataset, even better than FWNNN-DPC utilizing both contributions NLD and NLD together.

The results depicted in Table 9 also demonstrate that the capability of the NLD innovation even conceal that of the other innovation of NAS in FWNNN-DPC on 6/8 datasets including Target, Artil, Zelnik1, Complex9, Cdataset1 and Ellipse datasets. However, on Zelnik1 dataset, the single NAS innovation did significant contribution to enhance the performance of the base algorithm FKNN-DPC. Although the contribution NLD has also enhanced the performance of the baseline algorithm FKNN-DPC to some extent, both contributions NLD and NAS together cannot further enhanced the performance of FKNN-DPC, even inferior to that utilizing only one contribution NAS.

To the summary, the innovations NLD and NAS proposed in FWNNN-DPC enhanced the performance of the algorithm, resulting in the superiority of the FWNNN-DPC algorithm proposed in this paper over the peers in comparison.

## Conclusions

This paper proposes a new parameter-free density peak clustering algorithm, referred to FWNNN-DPC. It is founded on our enhanced natural nearest neighborhood and fuzzy weighted natural nearest neighbors. This FWNNN-DPC can find the natural nearest neighbors of a point automatically, so it does not require any parameters when defining the local density of a point not like DPC and its variants did.

Furthermore, the local density of a point, as defined in this paper, has the capacity to more readily identify the cluster centres of various density clusters, thereby significantly enhancing the accuracy of the clustering. Concurrently, the FWNNN assigns points to the most appropriate clusters, employing the shortest distance principle and the fuzzy weighted natural nearest neighbour relationships in the two-step assignment strategy, respectively. This further ensures the accuracy of the clustering.

Extensive experiments on both synthetic and real-world datasets demonstrate the superior performance of the FWNNN-DPC over its counterparts. Nevertheless, it should be noted that the detection of cluster centers is still performed manually, as was the case with the DPC. The identification of density peaks (i.e. cluster centres) within a dataset without human intervention remains a challenging problem that requires further attention. Furthermore, the adaptability of this FWNNN-DPC algorithm on high-dimensional datasets such as images or text embeddings need further investigating, which is our currently study.

## Data Availability

Most of the datasets can be accessed through references cited in this study. The Cdataset1, Ellipse and Artil datasets can be generated using the parameters provided in the related tables within this article.
